# The relationship between irritability and suicidal ideation: the chain mediating effects of rumination and distress tolerance

**DOI:** 10.1186/s12888-025-07598-2

**Published:** 2025-11-27

**Authors:** Kaimin Yao, Xiaojie Huang, Jingxiao Kuang, Mingzhi Xu

**Affiliations:** 1 Guangdong Mental Health Center, Guangdong Provincial People’s Hospital (Guangdong Academy of Medical Sciences), Southern Medical University, Guangzhou, Guangdong, 510120 China; 2 School of Public Health, Southern Medical University, Guangzhou, Guangdong, 510515 China

**Keywords:** Adolescent, Major depressive disorder, Irritability, Suicidal ideation, Rumination, Distress tolerance

## Abstract

**Background:**

Irritability is a core symptom of Major Depressive Disorder (MDD) in children and adolescent. Previous studies have reported a positive association between irritability and suicidal ideation. Based on the cognitive model of suicide and emotion regulation theory, the current study aimed to explore the role of rumination and distress tolerance in the relationship between irritability and suicidal ideation in adolescent patients with MDD.

**Methods:**

A cross-sectional survey was conducted with 319 adolescent patients with MDD. Patients were clinically interviewed using the Development and Well-Being Assessment (DAWBA). The Children’s Depression Inventory (CDI), Affective Reactivity Index (ARI), Ruminative Response Scale (RRS), Distress Tolerance Scale (DTS) and Positive and Negative Suicide Ideation Inventory (PANSI) were used to measure depressive severity, irritability, rumination, distress tolerance, and suicidal ideation. Descriptive and correlation analysis were conducted to examine the initial relationships among the main variables. Structural Equation Modeling (SEM) was conducted to investigate the mediating effects of rumination and distress tolerance in the relationship between irritability and suicidal ideation.

**Results:**

82.76% of adolescent patients with MDD reported experiencing irritability. Both rumination (*β* = 0.080, *p* < 0.001, 95% CI [0.037, 0.136]) and distress tolerance (*β* = 0.081, *p* < 0.001, 95% CI [0.033, 0.145]) independently mediated the relationship between irritability and suicidal ideation. And the chain mediating effects of rumination and distress tolerance in the relationship between irritability and suicidal ideation were significant (*β* = 0.048, *p* < 0.001, 95% CI [0.022, 0.086]).

**Conclusions:**

Rumination and distress tolerance played a chain mediating roles in the relationship between irritability and suicidal ideation, suggesting that rumination and distress tolerance may serve as important targets for intervention in the prevention and treatment of irritability and suicidal ideation in adolescents with MDD from an emotion regulation perspective.

**Clinical trial number:**

Not applicable.

## Background

Suicide is a serious public health issue worldwide. According to the World Health Organization’s report, suicide is the third leading cause of death among adolescent aged 15–19 years [1]. A population-based study among adolescents reported an overall prevalence of 16.9% (95% CI 15.0–18.8) for suicidal ideation, 17.0% (95% CI 14.8–19.2) for suicide planning, and 17.0% (95% CI 14.7–19.3) for suicide attempts [2]. Suicide is strongly associated with adolescents with mood disorders [3, 4]. Major depressive disorder (MDD) has been identified as the leading cause of adolescent suicidal behavior [5]. Depression is estimated to occur among 1.4% of adolescents aged 10–14 years, and 3.5% of 15–19 years old [6]. Alarmingly, a large-scale study in China found that the incidence of suicidal ideation among adolescent patients with depression was 38.23% [7]. Suicidal ideation refers to thoughts or plans about ending one’s life [8], it is a critical predictor of suicide attempt and completed suicide [9], suggesting that it is important to pay attention to suicidal ideation in adolescent patients with MDD. MDD is characterized by symptoms such as depressed mood, diminished interests, impaired cognitive function and vegetative symptoms [10]. An important distinguishing feature of adolescent MDD compared to adult MDD is irritability [11]. Irritability is listed as one of the core symptoms of MDD in children and adolescent in the fifth edition of the Diagnostic and Statistical Manual (DSM-5) [12].

According to the DSM-5, irritability is clinically manifested through frequent temper outbursts [13]. A systematic review reported that irritability as a transdiagnostic risk factor for suicidal behaviors, with irritable adolescents potentially being more prone to suicide attempts when experiencing suicidal ideation [14]. Several studies have found a positive association between irritability and suicidal ideation [15–17]. Orri et al. found that increasing irritability in childhood were closely related to suicide risk, and it appears to be a distal marker of suicidality acting through more proximal depressive severity [18]. The effect of irritability on suicidal ideation, however, is not simply direct; it is further influenced by other factors, such as internalizing/externalizing symptoms [14, 18], peer victimization [19] and hope [20]. Wenzel and Beck (2008) proposed a cognitive theory of suicide, which outlines three main constructs that underlie suicidal behavior from a cognitive perspective: dispositional vulnerability factors, cognitive processes associated with psychiatric disturbance and cognitive processes associated with suicidal acts [21]. The interaction of these three constructs may lead to suicidal ideation and behavior. As irritability is considered an important dispositional vulnerability factor [22], exploring its relationship with cognitive processes in MDD and suicidal ideation is crucial. These cognitive processes include negative cognitive biases, maladaptive emotion regulation strategies, and emotion regulation abilities [10, 23].

A common negative cognitive symptom in patients with MDD is rumination [24]. Rumination conceptualized as an avoidant emotion regulation strategy [25]. It involves individuals repeatedly and passively focusing on their negative emotions and the potential causes and consequences of these emotions after experiencing a negative life event [26]. Rumination is considered an important risk factor for the onset, severity and recurrence of MDD [27–29]. Longitudinal studies have found bidirectional associations between rumination and depressive symptoms [30], and higher levels of rumination in adolescents have been linked to a greater likelihood of developing MDD [31]. Increasing evidence suggests that rumination plays a pivotal role in the onset and worsening of suicidal ideation [32–35]. A 12-month longitudinal study in China found a significant association between high levels of rumination and suicidal ideation and attempts in patients with MDD [36]. Leigh et al. found that angry rumination was significantly associated with irritability six months later in a prospective study [37]. A longitudinal study of adolescents aged 12–20 years revealed a strong and stable correlation between irritability and hostile rumination over time [38]. Previous research has also established a significant association between rumination and distress tolerance [39, 40].

Distress tolerance is a critical emotion regulation ability defined as the capacity to experience and withstand negative psychological states [41]. It is a multidimensional construct encompassing tolerability and aversiveness, appraisal and acceptability, regulation of emotion, absorption of attention, and disrupt functioning [41]. Low distress tolerance is recognized as a key feature in the onset and maintenance of depression [42]. Related studies found that lower distress tolerance was significantly associated with greater suicidal ideation [43, 44]. A review suggested that individuals with poor tolerating distress tend to experience more severe depressive symptoms [42], and patients with MDD exhibit lower distress tolerance compared to non-depressed individuals [45]. Our previous research found that distress tolerance negatively predicts suicide risk in patients with MDD [46]. Yeşiloğlu et al. reported a close relationship between distress tolerance and suicidal ideation in MDD patients, with tolerance of physical pain partially mediating this relationship [47]. A case-control study also found that patients with MDD had enhanced irritability and poor distress tolerance compared with healthy controls [45]. Jeffries et al. (2016) investigated the relationship between four emotion regulation strategies (suppression, avoidance, rumination, and reappraisal) and distress tolerance, finding that distress tolerance was negatively correlated with suppression, avoidance, and rumination, but positively correlated with reappraisal [48]. Additionally, Sedighi et al. (2021) found that experiential avoidance and rumination negatively predicted distress tolerance [49].

Rumination is conceptualized as an avoidant emotional regulation strategy [25], while low distress tolerance is considered a maladaptive emotion regulation ability [41]. The relationship between emotional regulation strategies and abilities is bidirectional. Repeated use of maladaptive emotional regulation strategies may impair emotion regulation abilities over time [50]. A meta-analysis on emotional regulation strategies further indicated that severe rumination to some extent impairs an individual’s ability to distress tolerance [51]. Based on this theoretical background, we aimed to explore the role of rumination and distress tolerance in the relationship of irritability and suicidal ideation among adolescent patients with MDD. We hypothesized the following: (1) Irritability is positively associated with suicidal ideation; (2) Rumination mediates the relationship between irritability and suicidal ideation; (3) Distress tolerance mediates the relationship between irritability and suicidal ideation; (4) Rumination and distress tolerance play a chain mediating role in the relationship between irritability and suicidal ideation.

## Methods

### Study design and setting

A cross-sectional survey was conducted among 319 adolescent patients with MDD who were recruited from Guangdong Mental Health Center in Guangzhou, Guangdong province, China, from December 2023 to November 2024.

### Participants

All patients in this study met the following inclusion criteria: (1) a diagnosis of MDD according to the DSM-5; (2) aged between 12 and 18 years; (3) ability to understand and complete the questionnaires; (4) guardians and patients voluntarily agreed to participate in this study and signed the informed consent form. Patients were excluded if they met any of the following criteria: (1) any other psychiatric diagnoses according to DSM-5 diagnostic criteria; (2) a history of alcohol or substance abuse; (3) a history of organic brain disease and major craniocerebral injury; (4) having received modified electric convulsive therapy (MECT) in the past six months because MECT may induce transient cognitive disturbances, particularly memory impairment [52], which could compromise the reliability of self-reported questionnaire responses. To calculate the required sample size, we utilized the following sample size calculation formula *n* = [Z_1-α/2_^2*π(1-π)]/δ^2, which allowed for an absolute error δ = 0.1 and Z_1-α/2_ = 1.96. According to previous research, the prevalence of depression in Chinese adolescent was 38.23%, so π = 38.23% [7]. The minimum sample size in this study was calculated as *n* = [1.96^2^ × 38.23%×(1–38.23%)]/0.1^2^ ≈ 91. Therefore, the sample size in this study met the necessary requirements. This study was reviewed and approved by the Clinical Research Ethics Committee of Guangdong Provincial People’s Hospital (No. KY2024-262–02) and complied with the Declaration of Helsinki. Clinical trial number: not applicable.

### Procedure

Participants were recruited from the Guangdong Mental Health Center among adolescents aged 12–18 years diagnosed with MDD. Firstly, diagnoses were confirmed according to the DSM-5 by two attending psychiatrists or higher-level specialists. Secondly, a trained investigator introduced the study’s objectives and procedures to the patients. The study instructions and necessary precautions were read aloud to ensure understanding before the assessments. Following this, the Development and Well-Being Assessment (DAWBA) was administered for clinical interviews to assess symptom severity and confirm eligibility based on the predefined inclusion and exclusion criteria. Thirdly, all the participants and their parents or legal guardians signed the informed consent. Finally, the investigator collected sociodemographic information, and patients independently completed the self-report scales on-site. The interview and psychological evaluation were conducted in an undisturbed space and took about 40 minutes on average, and the questionnaires were collected by the investigator on the spot after completion.

## Measurements

### Development and well-being assessment

The Development and Well-Being Assessment (DAWBA) is a diagnostic assessment that complements the ICD-10 and DSM-IV diagnostic criteria for child and adolescent mental disorders [53]. It includes structured questions on symptoms and impairment, as well as open-ended qualitative questions. To reduce participant burden, only the depression section of the DAWBA was used to assess MDD in this study. This section consists of two parts: 24 structured questions and 11 semi-structured open-ended questions. The depression section covers four subsections: depression, irritability, loss of interest, and self-harm. The DAWBA has been shown to have good reliability and validity in Chinese adolescents with MDD (Kappa = 0.83, *P*＜0.001) [54].

### Depressive severity

The Children’s Depression Inventory (CDI) was used to assess depressive severity in adolescent patients with MDD over the past two weeks [55]. The CDI includes 27 items across five subscales: anhedonia, negative mood, negative self-esteem, inefficiency, and interpersonal problem. Each item is rated on a 3-point Likert scale (0–2), with total scores ranging from 0 to 54. Higher scores indicate greater depressive severity. The Chinese version of the CDI has demonstrated good reliability and validity in Chinese adolescents, with a Cronbach’s α coefficient of 0.88 [56]. In this study, the Cronbach’s α coefficient of CDI was 0.814, with skewness of −0.162 and kurtosis of −0.053.

### Irritability

The Affective Reactivity Index (ARI) was used to assess irritability levels in adolescent patients with MDD [57]. The ARI consists of six symptom items and one impairment item. Each symptom item is rated on a 3-point Likert scale (0–2), with the total score ranging from 0 to 12. Higher scores indicate higher levels of irritability. The impairment item is not included in the total score. The Chinese version of the ARI has been shown to be a reliable and valid tool for assessing irritability in Chinese adolescents, with a Cronbach’s α coefficient of 0.81 [58]. In this study, the Cronbach’s α coefficient of ARI was 0.808, with skewness of −0.887 and kurtosis of 0.362.

### Rumination

The Ruminative Response Scale (RRS) was used to assess rumination in adolescent patients with MDD [59]. The RRS includes 22 items across three subscales: symptom rumination, brooding, and reflective pondering. Each item is rated on a 4-point Likert scale (1 = never, 4 = always), with total scores ranging from 22 to 88. Higher scores indicate greater rumination. The Chinese version of the RRS has demonstrated good psychometric properties, with a Cronbach’s α coefficient of 0.93 [60]. In this study, the Cronbach’s α coefficient of RRS was 0.905, with skewness of −0.368 and kurtosis of −0.427.

### Distress tolerance

The Distress Tolerance Scale (DTS) was used to assess the ability to experience and withstand negative psychological states [41]. The Chinese version of the DTS has shown good reliability with a Cronbach’s α coefficient of 0.91 [61]. The 15-item DTS includes four factors: tolerance, absorption, appraisal, and regulation. Responses are rated on a 5-point Likert scale (1 = strongly agree, 5 = strongly disagree), with higher scores reflecting better distress tolerance. In this study, the Cronbach’s α coefficient of DTS was 0.808, with skewness of 0.593 and kurtosis of 0.697.

### Suicidal ideation

The Positive and Negative Suicide Ideation Inventory (PANSI) was used to assess suicidal ideation in adolescent patients with MDD over the past two weeks [62]. It includes the 8-item negative suicidal ideation (PANSI-NSI) and the 6-item positive suicidal ideation (PANSI-PSI). Each item is rated on a 5-point Likert scale (1 = none of the time, 5 = most of the time), with total scores ranging from 14 to 70. Higher scores indicate greater severity of suicidal ideation. The Chinese version of PANSI has been widely used in Chinese adolescent populations, with a Cronbach’s α coefficient of 0.86–0.94 [63, 64]. In this study, the Cronbach’s α coefficient of PANSI was 0.896, with skewness of −0.320 and kurtosis of −0.419.

### Statistical analysis

Statistical analyses were performed using SPSS 26.0 [65] and AMOS Version 24.0 [66]. Firstly, descriptive analysis summarized sociodemographic characteristics, and Pearson’s correlation analysis examined relationships between main variables (depressive severity, irritability, rumination, distress tolerance, suicidal ideation). Secondly, we compared differences in sociodemographic characteristics and main variables between irritability and non-irritability group. To be specific, continuous variables were performed using independent sample t-test and Mann-Whitney U t-test. Chi-square test was used to compare group differences among categorical variables. Thirdly, we adopted structural equation modeling (SEM) to examine the mediating role of rumination and distress tolerance in the relationship between irritability and suicidal ideation. The standards of goodness of fit indices included: ratio of Chi-square to the degree of freedom (χ^2^/df) should be less than 3, the Normed Fit Index (NFI) ≥ 0.90, the Comparative Fit Index (CFI) ≥ 0.90, the Goodness of Fit Index (GFI) ≥ 0.90, the Tucker-Lewis Index (TLI) ≥ 0.90, the Root Mean Square of Approximation (RMSEA) ≤ 0.08 [67]. Irritability was analyzed as the independent variable, suicidal ideation as the dependent variable, rumination and distress tolerance as the mediator, gender and currently receiving medication and/or psychotherapy were as the control variables. Bootstrap with 5000 iterations was used to calculate the 95% bias-corrected bootstrap confidence intervals of direct and indirect effects.

## Results

### Descriptive statistics of the sample characteristics and comparison of differences in suicide ideation

Detailed descriptions and comparison of sample were presented in Table 1. A total of 319 adolescent patients with MDD were analyzed, including 91 boys (28.53%) and 228 girls (71.47%). The mean age of the sample was (15.14 ± 1.65) years and the mean years of education was (9.30 ± 1.69) years. 308 patients (96.55%) were Han nationality, 171 patients (53.61%) were urban, 62 patients (19.44%) were only children, 40 patients (12.54%) were left-behind children, 52 patients (16.30%) were divorcing family. Most of patients (68.34%) lived with their parents. 36 patients (11.29%) had a family history of mental disorder, 36 patients (11.29%) had physical disease, and 212 patients (66.46%) were currently receiving medication and/or psychotherapy. A total of 272 patients (85.27%) were first-episode, 43 patients (13.48%) reported one previous episode, while only 4 (1.25%) reported two. The mean age of first onset was (13.96 ± 1.89) years and the mean course of MDD was (16.89 ± 13.97) months. According to DWABA interview results, there were 264 adolescent patients with MDD had irritability (82.76%). By comparing characteristics in suicidal ideation, we found a significant gender difference in suicidal ideation (*t* = −2.557, *p* < 0.05) (Table 1).

**Table 1:** Socio-demographic characteristics of the sample and comparison of differences in suicide ideation (*n* = 319)

Variables		**t/χ**^**2**^**/Z**
**Age, M(SD)**	15.14 ± 1.65	
**Years of education, M(SD)**	9.30 ± 1.69	
**Gender, cases (%)**		−2.557^*^
Male	91 (28.53%)	
Female	228 (71.47%)	
**Nation, cases (%)**		−0.871
Han nationality	308 (96.55%)	
Ethnic minority	11 (3.45%）	
**Residence, cases (%)**		−0.401
Rural	148 (46.39%)	
Urban	171 (53.61%)	
**Only child, cases (%)**		−0.226
Yes	62 (19.44%)	
No	257 (80.56%)	
**Left-behind children, cases (%)**		0.752
Yes	40 (12.54%)	
No	279 (87.46%)	
**Divorcing family, cases (%)**		1.494
Yes	52 (16.30%)	
No	267 (83.70%)	
**Living with, cases (%)**		0.743
Alone	10 (3.13%)	
Parent	218 (68.34%)	
Father	14 (4.39%)	
Mother	47 (14.73%)	
Grandparent	24 (7.52%)	
Other people	6 (1.88%)	
**A family history of mental disorder, cases (%)**		1.293
Yes	36 (11.29%)	
No	283 (88.71%)	
**Physical disease, cases (%)**		0.321
Yes	36 (11.29%)	
No	283 (88.71%)	
**Currently receiving medication and/or psychotherapy, cases (%)**		0.093
Yes	212 (66.46%)	
No	107 (33.54%)	
**First-onset, cases (%)**		−1.488
Yes	272 (85.27%)	
No	47 (14.73%)	
**Number of previous episodes: 0, cases (%)**	272 (85.27%)	
**Number of previous episodes: 1, cases (%)**	43 (13.48%)	
**Number of previous episodes: 2, cases (%)**	4 (1.25%)	
**Age of onset, M(SD)**	13.96 ± 1.89	
**Course of MDD (months), M(SD)**	16.89 ± 13.97	

Note: M, Mean; SD, standard deviation; N, Median; MDD, major depressive disorder; significance was set as ^*^ *p* < 0.05; ^**^ *p* < 0.01; ^***^ *p* < 0.001

### Correlation analysis in main clinical variables in patients with MDD

Depressive severity was positively correlated with irritability (*r* = 0.351, *p* < 0.001), rumination (*r* = 0.544, *p* < 0.001) and suicidal ideation (*r* = 0.694, *p* < 0.001). Irritability was positively correlated with rumination (*r* = 0.197, *p* < 0.001) and suicidal ideation (*r* = 0.278, *p* < 0.001). Suicidal ideation was positively correlated with rumination (*r* = 0.421, *p* < 0.001). And distress tolerance was negatively correlated with depressive severity (*r* = −0.459, *p* < 0.001), irritability (*r* = −0.264, *p* < 0.001), rumination (*r* = −0.400, *p* < 0.001), and suicidal ideation (*r* = −0.433, *p* < 0.001). Correlations among the main variables were summarized in Table 2.

**Table 2:** Correlation analysis, mean and standard deviation among the main variables (*n* = 319)

Variables	Mean ± SD	1	a	b	c	d	e	2	3	4	5
**1. Depressive severity**	32.03 ± 7.08	-	-	-	-	-	-	-	-	-	-
**a. Anhedonia**	9.78 ± 2.55	0.805^***^	-	-	-	-	-	-	-	-	-
**b. Negative Mood**	6.92 ± 2.18	0.780^***^	0.557^***^	-	-	-	-	-	-	-	-
**c. Negative Self-Esteem**	6.52 ± 1.91	0.741^***^	0.418^***^	0.453^***^	-	-	-	-	-	-	-
**d. Ineffectiveness**	5.30 ± 1.52	0.623^***^	0.321^***^	0.362^***^	0.431^***^	-	-	-	-	-	-
**e. Interpersonal Problem**	3.50 ± 1.47	0.659^***^	0.445^***^	0.349^***^	0.430^***^	0.316^***^	-	-	-	-	-
**2. Irritability**	7.40 ± 2.95	0.351^***^	0.248^***^	0.328^***^	0.200^***^	0.231^***^	0.277^***^	-	-	-	-
**3. Rumination**	65.45 ± 12.12	0.544^***^	0.488^***^	0.496^***^	0.301^***^	0.338^***^	0.298^***^	0.197^***^	-	-	-
**4. Distress tolerance**	36.55 ± 9.17	−0.459^***^	−0.370^***^	−0.367^***^	−0.338^***^	−0.360^***^	−0.215^***^	−0.264^***^	−0.400^***^	-	-
**5. Suicidal ideation**	50.70 ± 10.31	0.694^***^	0.431^***^	0.530^***^	0.719^***^	0.461^***^	0.399^***^	0.278^***^	0.421^***^	−0.433^***^	-

Note: significance was set as ^*^ *p* < 0.05; ^**^ *p* < 0.01; ^***^ *p* < 0.001

### Mediation analyses of rumination and distress tolerance on the relationship between irritability and suicidal ideation

**Fig. 1:**
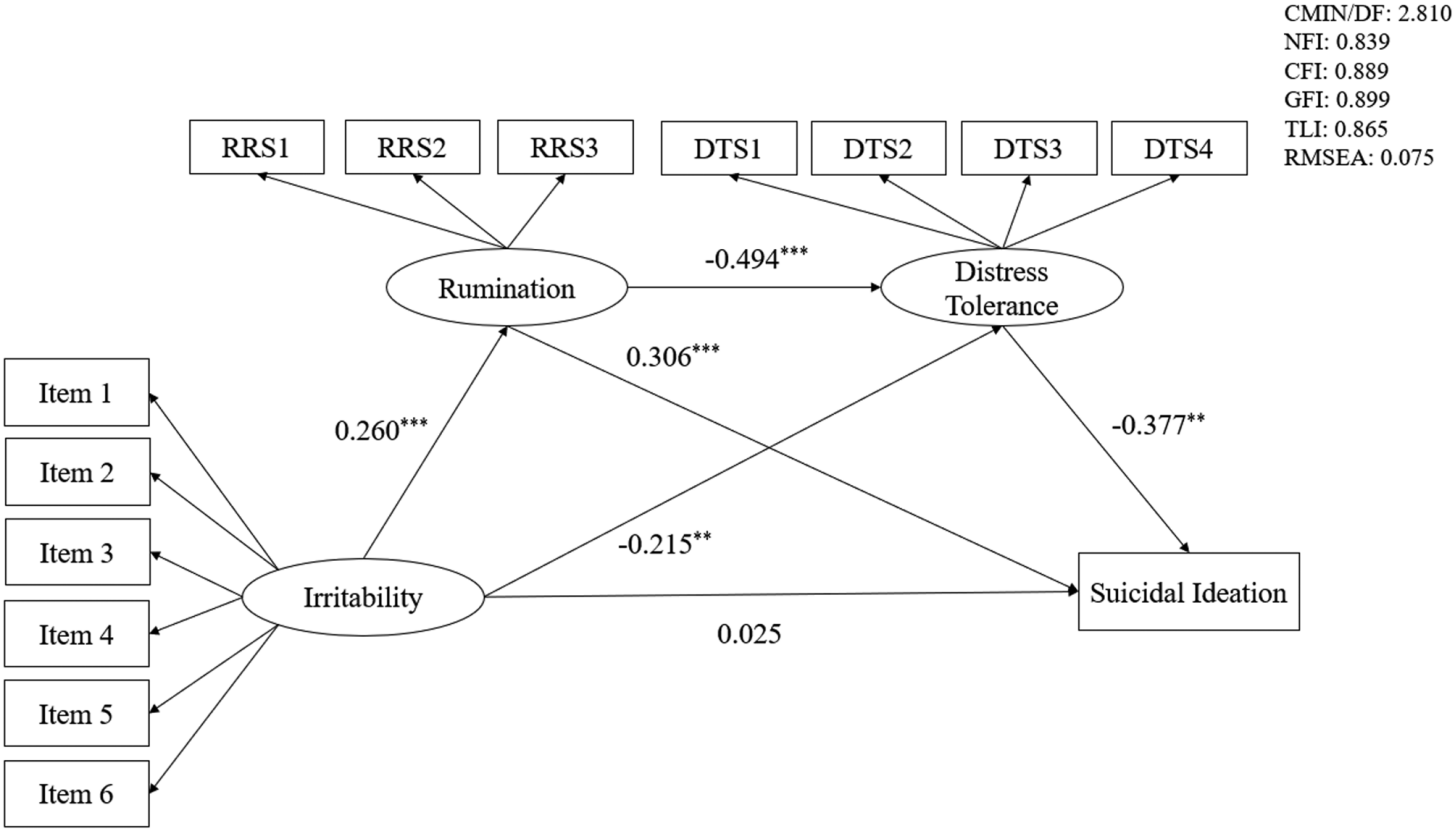
Rumination and distress tolerance as mediators in the relationship between irritability and suicidal ideation. Note: gender and Currently receiving medication and/or psychotherapy were used as control variables; significance was set as ^*^ *p* < 0.05; ^**^ *p* < 0.01; ^***^ *p* < 0.001; RRS1, symptom rumination; RRS2, reflective pondering; RRS3, brooding; DTS1, tolerance; DTS2, absorption; DTS3, appraisal; DTS4, regulation; CMIN/DF, ratio of Chi-square to the degree of freedom; NFI, the Normed Fit Index; CFI, the Comparative Fit Index; GFI, the Goodness of Fit Index; TLI, the Tucker-Lewis Index; RMSEA, the Root Mean Square of Approximation; CMIN/DF: 2.898; NFI: 0.855; CFI: 0.899; GFI: 0.902; TLI: 0.875; RMSEA: 0.077

We tested the mediation model using SEM. The model produced acceptable fit indices (χ^2^/df = 2.810, *p* < 0.001, RMSEA = 0.075, NFI = 0.839, CFI = 0.889, GFI = 0.899, TLI = 0.865). Figure 1 showed the regression coefficients for the mediation model of irritability to suicidal ideation through rumination and distress tolerance. Specifically, the paths from irritability to rumination (*β* = 0.260, *p* < 0.001) and from rumination to suicidal ideation (*β* = 0.306, *p* < 0.001) were significant. The indirect effect of irritability on suicidal ideation through rumination was found to be significant (*β* = 0.080, *p* < 0.001, 95% CI [0.037, 0.136]), which accounted for 34.19% of the total effect. Besides, the paths from irritability to distress tolerance (*β* = −0.215, *p* < 0.01) and from distress tolerance to suicidal ideation (*β* = −0.377, *p* < 0.01) were significant. The indirect effect of irritability on suicidal ideation through distress tolerance also was found to be significant (*β* = 0.081, *p* < 0.001, 95% CI [0.033, 0.145]), which accounted for 34.62% of the total effect. Moreover, the current results also revealed that the chain mediating effects of rumination and distress tolerance in the relationship between irritability and suicidal ideation were significant (*β* = 0.048, *p* < 0.001, 95% CI [0.022, 0.086]), which accounted for 20.00% of the total effect. And rumination could negatively predict distress tolerance (*β* = −0.494, *p* < 0.001). Detailed effect sizes of direct and indirect paths were presented in Table 3.

**Table 3:** The bootstrap analysis of paths and effects (*n* = 319)

Effect	Path	Effect size			
		Effect	SE	95% CI	Ratio of effect
**Direct effect**	Irritability → Suicidal ideation	0.025	0.055	(−0.082, 0.135)	10.68%
**Indirect effect**	Irritability → Rumination → Suicidal ideation	0.080	0.024	(0.037, 0.136)	34.19%
	Irritability → Distress tolerance → Suicidal ideation	0.081	0.028	(0.033, 0.145)	34.62%
	Irritability → Rumination → Distress tolerance → Suicidal ideation	0.048	0.016	(0.022, 0.086)	20.51%
**Total effect**		0.234	0.058	(0.118, 0.345)	

Note: SE, standard error; 95% CI, 95% bias – corrected Confidence Interval, Gender and Currently receiving medication and/or psychotherapy were used as control variables

## Discussion

The current study aimed to explore the effects of rumination and distress tolerance in the relationship of irritability and suicidal ideation among adolescent patients with MDD. Our findings found that most of adolescent patients with MDD (82.76%) was irritable, providing further evidence that irritability is a core symptom of MDD in this population. An integrative review suggested that 44% to 82% of individuals experience low and stable levels of irritability from early childhood to early adolescence [68]. This study also found that suicidal ideation was significantly higher in girls than boys. Gender is an important factor in suicidal behavior, with higher rates of ideation and suicide attempts among women [69]. Furthermore, we found a significant positive correlation between irritability and suicidal ideation, indicating that higher levels of irritability in adolescents with MDD are associated with more severe suicidal ideation.

As hypothesized, the present findings showed that rumination independently mediated the relationship between irritability and suicidal ideation. Higher levels of irritability in adolescent patients with MDD were associated with more severe rumination, which, in turn, significantly contributed to the intensity of suicidal ideation. Rogers et al. suggested that rumination could serve as a potential catalyst linking affective states (e.g., irritability, depression, anxiety) to suicidal ideation [33]. As a maladaptive emotion regulation strategy, excessive rumination interferes with effective problem-solving, leading to impaired emotional regulation, which may increase the frequency of suicidal ideation [70]. Individuals prone to focusing on their distress and related thoughts may be more likely to experience recurrent suicidal ideation in response to negative emotions and experiences [37].

And we also found that distress tolerance independently mediated the relationship between irritability and suicidal ideation. Previous studies suggested that irritability is associated with heightened frustration beyond initial reactivity and inhibitory control weaknesses, which are linked to the ability to tolerate distress [71]. Ellis et al. found that MDD patients with low distress tolerance have difficulty regulating naturally occurring anger and correspondingly have higher levels of irritability [45]. Pompili et al. found that patients reporting severe depressive symptoms and high mental pain presented a mixture of particular dangerousness, characterized by high trait hopelessness, more frequent and less controllable suicidal ideation, and previous suicide behaviors [72]. Relevant studies have confirmed the relationship between distress tolerance and suicidal ideation [43, 44, 73, 74]. Irritable adolescents with MDD overreact to stimuli and experience more intense negative emotions when facing setbacks [75]. These individuals may struggle to regulate their negative emotions effectively when such emotions exceed their distress tolerance capacity, leading them to view suicide as a potential solution to unbearable emotional distress [76].

Moreover, the current study found that rumination and distress tolerance played a significant chain mediating role between irritability and suicidal ideation, showing that irritability can influence their distress tolerance through rumination, further contributing to suicidal ideation. And rumination could negatively predict distress tolerance, which means having too much rumination maybe reduce patients’ distress tolerance. Previous studies have found a significant association between more serious rumination and lower distress tolerance [39, 40, 48, 49]. The relationship between emotion regulation abilities and strategies is bidirectional, and repeated use of maladaptive emotion regulation strategies may lead to a reduction in certain emotion regulation abilities [50]. A meta-analysis confirmed the detrimental effect of severe rumination on distress tolerance [51]. According to the cognitive theory of suicide [21], irritability acts as a dispositional vulnerability factor [22], rumination is a cognitive process associated with MDD [25], and distress tolerance is associated with suicidal acts [41]. The combined effect of serious rumination and low distress tolerance would highly increase suicidal ideation in irritable adolescent patients with MDD. Our results integrate emotion regulation strategies and abilities to explain the relationship between irritability and suicidal ideation in adolescents, suggesting that rumination and distress tolerance should be key targets for intervention in the prevention and treatment of irritability and suicidal ideation among adolescents with MDD.

Irritability is one of the most widely recognized transdiagnostic constructs in the DSM-5, covering 15 mental disorders [77]. Copeland et al. (2015) categorized irritability into tonic irritability and phasic irritability based on its duration [78]. Leibenluft et al. (2006) differentiated chronic irritability from episodic irritability, based on duration and the different expressions of irritability [79]. In this study, irritability was primarily assessed as a trait-like construct using the ARI, which captures tendencies toward chronic, tonic irritability—characterized by frequent temper outbursts [57]. While there is a substantial body of research on irritability abroad, studies on adolescent irritability in China remain limited. This study is the first to explore the relationship between irritability and suicidal ideation in Chinese adolescents, which may have significant implications for future research and psychotherapeutic applications. On one hand, targeted interventions could be implemented to reduce negative thoughts associated with rumination, effectively reducing suicidal ideation in irritable adolescents. Previous studies have shown that cognitive behavioral therapy, metacognitive therapy, and mindfulness training are beneficial for improving rumination [80–82]. On the other hand, distress tolerance training for adolescent patients with MDD could be strengthened to enhance emotional regulation in irritable moods, which may help reduce suicidal ideation. Studies have found that mindfulness therapy and dialectical behavior therapy can effectively improve distress tolerance [83, 84].

## Clinical implications

The findings of the current study will be valuable for clinical decision-making and psychological care for adolescent patients with MDD. These results provide an empirical foundation for developing individualized interventions. Clinicians and therapists can incorporate emotion regulation training, cognitive restructuring, and distress tolerance skill-building into treatment plans. Multi-target strategies could help reduce irritability, improve cognitive flexibility, and enhance the prevention and management of suicidal ideation in adolescent patients with MDD.

## Limitations

There were still some limitations in this study. Firstly, a convenience sampling method was used to recruit participants, which may have resulted in a sample that is not fully representative, potentially affecting the generalizability of the findings. Secondly, recruiting participants from a single-center limits the external validity of the study, so future multi-center studies could provide more robust conclusions. And our findings may not generalize to adolescent with co-occurring substance use disorders, as such individuals were excluded from the study sample. Thirdly, the PANSI measures suicide-related constructs (e.g., hopelessness, problem-solving deficits) rather than directly assessing suicidal ideation, which may limit the granularity of our findings regarding this specific outcome. Additionally, while we focused on trait irritability, which is relevant for identifying individuals with stable vulnerabilities, we acknowledge the importance of state irritability. Phasic or episodic irritability, measured in real-time or following specific triggers, may offer unique insights into dynamic processes leading to acute clinical outcomes, such as suicidal crises. Future research should include measures of state irritability to better understand how momentary fluctuations interact with trait vulnerabilities to influence acute behavioral outcomes. Finally, given that this study used a cross-sectional design, no causal conclusions can be drawn. Longitudinal or cohort studies would be necessary to further explore the mechanisms linking irritability and suicidal ideation.

## Conclusions

Our study found that rumination and distress tolerance played a chain mediating role in the relationship between irritability and suicidal ideation in adolescent patients with MDD. These findings suggest that rumination and distress tolerance may serve as important targets for intervention in the prevention and treatment of irritability and suicidal ideation in adolescents with MDD from an emotion regulation perspective.

## Data Availability

Due to ethical restrictions, the present study data were not publicly available to ensure that research participants privacy is not compromised. Data for this study are available from the corresponding author, Dr. Xu.

## Abbreviations

MDD: Major Depressive Disorder
DAWBA: Development and Well-Being Assessment
CDI: Children’s Depression Inventory
ARI: Affective Reactivity Index
RRS: Ruminative Response Scale
DTS: Distress Tolerance Scale
PANSI: Positive and Negative Suicide Ideation Inventory
SEM: Structural Equation Modeling
95% CI: 95% bias–corrected Confidence Interval
DSM-5: The fifth edition of the Diagnostic and Statistical Manual
MECT: Modified Electric Convulsive Therapy
ICD-10: International Classification of Diseases 10th Revision
DSM-IV: The fourth edition of the Diagnostic and Statistical Manual
CMIN/DF: Ratio of Chi-square to the degree of freedom
NFI: Normed Fit Index
CFI: Comparative Fit Index
GFI: Goodness of Fit Index
TLI: Tucker-Lewis Index
RMSEA: The Root Mean Square of Approximation
M: Mean
SD: Standard Deviation
N: Median
RRS1: Symptom rumination
RRS2: Reflective pondering
RRS3: Brooding
DTS1: Tolerance
DTS2: Absorption
DTS3: Appraisal
DTS4: Regulation
